# High rates of parasite recrudescence following intermittent preventive treatment with sulphadoxine-pyrimethamine during pregnancy in Benin

**DOI:** 10.1186/1475-2875-12-195

**Published:** 2013-06-10

**Authors:** Azizath Moussiliou, Yolande Sissinto-Savi De Tove, Justin Doritchamou, Adrian JF Luty, Achille Massougbodji, Michael Alifrangis, Philippe Deloron, Nicaise Tuikue Ndam

**Affiliations:** 1 Institut de Recherche pour le Développement, UMR216 Mère et enfant face aux infections tropicales, Faculté des sciences biologiques et pharmaceutiques, 4, avenue de l’observatoire, Paris 75006, France; 2 Faculté de Pharmacie, Université Paris Descartes, PRES Sorbonne Paris Cité, Paris 75270, France; 3 Centre d’Etude et de Recherche sur le Paludisme associé à la Grossesse et à l’Enfance, Faculté des Science de Santé, Université d’Abomey-Calavi, Cotonou 01 BP 188, Benin; 4 Department of International Health, Immunology and Microbiology, Centre for Medical Parasitology Faculty of Health, Medical Sciences, University of Copenhagen, Copenhagen K 1014, Denmark; 5 Department of Infectious Diseases, Copenhagen University Hospital (Rigshospitalet), Copenhagen K 1014, Denmark

**Keywords:** Plasmodium falciparum, Pregnancy, IPTp, Sulphadoxine-pyrimethamine, Mutations, pfdhfr, pfdhps, Benin

## Abstract

**Background:**

Despite widespread parasite resistance to sulphadoxine-pyrimethamine (SP) its use for intermittent preventative treatment during pregnancy remains the policy in Benin and throughout most of sub-Saharan Africa.

**Methods:**

In a prospective study, 982 pregnant women were recruited in Benin and followed until delivery. The prevalence of point mutations in the *pfdhfr* and *pfdhps* genes associated with *Plasmodium falciparum* resistance to SP during consecutive antenatal visits was determined. Parasites clearance among women infected at SP intake was assessed by microscopy and PCR. Association between the persistence of parasites and malaria consequences, were investigated. Recurrent parasites were genotyped to identify recrudescences from re-infections.

**Results:**

The prevalence of *pfdhfr*/*pfdhps* quadruple mutants (triple *pfdhfr* + single *pfdhps*) was consistently above 80% while quintuple and sextuple mutants remained low. Importantly the higly mutated parasites apparently never included the two key mutations, *pfdhfr* 164 L or *pfdhps* 540E. Based on PCR results, SP failed to clear existing parasitaemia in half (48%) of the women who were infected at IPTp schedule. The frequency of recrudescence reached 76% after the second dose. Women with persistent parasitaemia had an increased prevalence of anaemia (*P = 0.03*).

**Conclusion:**

The data presented here, highlight the inability of SP to ensure optimal antiplasmodial protection in late pregnancy, and invite urgent consideration of an alternative drug or strategy.

## Background

Malaria is an important cause of maternal and perinatal morbidity and mortality [1]. Pregnancy-associated malaria (PAM) is characterized by the accumulation of *Plasmodium falciparum*-infected erythrocytes (PfIE) in placental intervillous spaces [2]. The occurrence of low birth weight (LBW) is one of the main consequences of PAM and this is associated with a high risk of neonatal mortality. Approximately 100,000 children die annually in sub-Saharan Africa due to malaria-related LBW [3].

In Benin, anti-malarial treatment policy has long been based on the use of chloroquine (CQ) as first-line and SP as second-line. CQ was also used in prophylaxis of malaria during pregnancy. However, decreased therapeutic efficacies of both CQ and SP prompted the change of the national anti-malarial drug policy in 2004 by the official withdrawal of CQ and SP in the treatment of uncomplicated malaria. As reported in many parts of the world sulphadoxine-pyrimethamine (SP) has been used extensively in the past for treatment and prophylaxis of falciparum malaria [4]. One of the consequences is that the utility of SP in the treatment of malaria in sub-Saharan Africa has declined drastically in the last decade because of the emergence and spread of drug resistance of *P. falciparum.* However, SP is still used for malaria prevention during pregnancy as intermittent preventive treatment in pregnancy (IPTp). The SP-IPTp regimen has been recommended by the World Health Organization (WHO) [5] since 2004, and was adopted by Benin the same year. SP-IPTp regimen comprises the administration of at least two curative doses of SP starting in the second trimester of pregnancy (16 weeks of gestation) and ensuring a dose spacing interval of at least one month. SP continues to be the drug of choice for IPTp both because it is safe, easy to administer, and because a single treatment dose has long-lasting prophylactic effect (up to 60 days) [6,7]. SP efficacy is dependent on the number of mutations accumulated in the genes encoding the *P. falciparum* enzymes dihydrofolate reductase (DHFR) and dihydropteroate synthase (DHPS) [8,9]. Single nucleotide polymorphisms (SNPs) in the *pfdhfr* gene, at codons 51, 59, 108, and 164, and in the *pfdhps* gene, at codons 436, 437, 540, 581, and 613 are associated with *in vitro* resistance to pyrimethamine and sulphadoxine, respectively [10-12]. Parasite resistance to SP *in vivo* is largely associated with a triple mutation in *pfdhfr* (resulting in amino acid changes; N51I, C59R and S108N) coupled with a double mutation in *pfdhps* (A437G, K540E) [13-15]. Such *P. falciparum* parasites carrying quintuple mutations (triple *pfdhfr* with double *pfdhps* mutations) are highly prevalent in East Africa [16,17], but rare in West Africa [18], including Benin where a study from 2003 to 2005 showed that the most prevalent haplotype (85%) was the quadruple (triple *pfdhfr*, single *pfdhps*) mutant [19]. Although SP shows poor efficacy in children infected with quintuple mutant parasites, IPTp with SP seems to remain effective in preventing the adverse consequences of malaria on maternal and foetal outcomes in areas where a high proportion of *P. falciparum* parasites carry these quintuple mutations [20,21].

From 2005 to 2007, a study in southern Benin [22] showed that the proportion of parasites carrying quadruple *pfdhfr*/*pfdhps* mutations during pregnancy was constantly above 80% whilst no quintuple mutants were detected. Recently, a prospective cohort study (STOPPAM) on ~1,000 pregnant women in south-western Benin, four years after the change of malaria treatment policy in Benin was conducted. A subset of samples collected during the follow up were used to assess the prevalence and possible selection of molecular markers of SP resistance in the *pfdhfr* and *pfdhps* genes present at different gestational ages, and to characterize recurrent infections in the context of SP-IPTp.

## Methods

### Study area

The study was conducted from 2008 to 2010 in Comé, a semi-rural area of southern Benin, 70 km west of Cotonou, the capital of Benin. Malaria transmission is perennial, with two peaks during the two rainy seasons. The entomological inoculation rate (EIR) ranges from 35 to 60 infective bites per person and per year [23], with *P. falciparum* predominating [24]. The study area has been described elsewhere [25].

### Study design, collection and handling of blood samples

The study comprised a cohort of pregnant women included before 24 weeks of pregnancy. Detailed descriptions of the pregnant women follow-up have been reported elsewhere [25]. Briefly, 982 women were recruited during the first antenatal visit and followed up monthly from inclusion to delivery. Two doses of SP-IPTp (IDA, The Netherlands) were administered following national guidelines. The exact gestational age was determined by using an ultrasound scans, performed with a portable ultrasound system (Titan Sonosite Bothell WA). A rapid diagnostic test (Parascreen™, Zephyr Biomedicals Goa, India) for identification of *P. falciparum* infection was performed on capillary blood. Venous blood was taken at inclusion, at each antenatal visit, and at unscheduled ‘emergency’ visits when women presented at the clinic for health reasons. At delivery, placental and peripheral blood was also collected. Thick and thin blood films were prepared from all blood samples to confirm active *P. falciparum* infection, Giemsa-stained, and read by two independent, experienced microscopists.

Four separate drops of blood were spotted onto Whatman 3MM filter paper and stored with silica gel before DNA extraction using the Chelex 100 resin method [26]. Samples from 212 women were used for *pfdhfr/pfdhps* genotyping. A subset of 107 samples based on the availability of samples from days 7 to 60 following the SP uptake was identified to assess drug efficacy. Isolates from parasitized women at each SP-IPTp administration (either first or second dose) and each sample of these women until next SP administration, or for a 60-day period were examined. Women having received anti-malarial treatment other than SP-IPTp during this follow up were excluded.

The study was approved by the ethics committees of the Research Institute for Development (IRD) in France and the Faculty of Health Science (University of Abomey-Calavi) in Benin. Written informed consent was given by all women participating in this study.

### Real-time PCR assay for the detection of *Plasmodium falciparum* infections

A duplex real-time PCR assay using genus-specific and species-specific primers and probes (*Plasmodium spp/P. falciparum*) for the gene encoding the small subunit (18S) of *Plasmodium* rRNA was used to screen samples containing parasites following treatment, as described [27]. Samples underwent 40 cycles of amplification using the ViiA™ 7 Real-Time PCR system (Applied Biosystem) and were quantified using a DNA standard range made from a suspension of *in vitro* cultured 3D7 *P. falciparum* line (obtained through the MR4 as part of the BEI Resources Repository, NIAID, NIH: *Plasmodium falciparum* 3D7 GL, MRA-1001, deposited by Megan Dowler, Walter Reed Army Institute of Research).

### Nested PCR amplification of *pfdhfr* and *pfdhps* genes and detection of SNPs

Parasite DNA was amplified with outer and nested specific primers targeting the *pfdhfr* and *pfdhps* genes, as described [28,29]. The SNPs in the *pfdhfr* and *pfdhps* genes were captured using sequence specific oligonucleotide probes-enzyme linked immunosorbent assay (SSOP-ELISA) technique, as described [29]. Briefly, 96-well PCR plates (Maxisorp, Nunc, Roskilde, Denmark) were coated with streptavidin and incubated overnight at +4°C. Nested PCR products were diluted (1:10), denatured at 95°C and immediately cooled at +4°C before being incubated on the plate. Individual digoxigenin-conjugated SSOPs probes targeting *pfdhfr* codons c50/51 (CI/CN), c59 (C/R), c108 (S/N/T) and c164 (I/L), and *pfdhps* codons c436/c437 (AA/AG/SA/SG/FG), c540 (K/E), c581 (A/G) and c613 (A/S) were added. Plates were incubated in a shaker-incubator (ES-20/60, Biosan, Latvia) followed by stringent washing with tetra-methyl-ammonium chloride (Sigma, Germany). Horseradish peroxidase-conjugated anti-digoxigenin antibody with o-phenylene-diamine tablets dissolved in hydrogen peroxide was used as substrate. The reaction was stopped by addition of sulphuric acid. Optical densities (OD) were read at 492 nm. For each SNP, samples were categorized into single or mixed infections. Single genotype infections harboured a single SNP present at OD values above the threshold of positivity. Mixed genotype samples contained a main SNP genotype and a minor genotype whose OD value was less than half (mixed with dominant genotype) or more than half (mixed with no dominant genotype) the OD value of the main genotype. For samples that contained infections categorized as single or mixed with a dominant SNP type at all analysed codons, results were combined to construct haplotypes.

### Molecular genotyping of the polymorphic genes *msp1* (block 2) and *msp2*

Analysis of MSP-2 (3D7 and FC27 allelic families) and MSP1 block 2 (K1, MAD20 and RO33 allelic families) of *P. falciparum* were sequentially performed by nested PCR in accordance with the genotyping protocol of Snounou [30]. DNA genotypes of samples collected at baseline (IPTp uptake) and on the day of recurrent infection were compared according to band size and number, for each of the allelic families of *msp1* block 2 and *msp2.* To differentiate new infections from recrudescent ones, gel photographs were scored by visual comparison of DNA fragments obtained from baseline and re-infection samples. Unamplified samples and those showing the same *msp2* profile on both consecutive samples were analysed on the *msp1* block 2 markers. An infection was considered to be a recrudescence when identical pre-treatment allele(s) were found in post-treatment samples, the two patterns being either completely identical or containing some missing or additional clones. Infections were considered as re-infections if the allelic pattern for any of the loci differed completely between the pre- and post-treatment samples.

### Data analysis

Data were analysed using STATA version 11 (Stata Corp, College Station, TX, USA). Descriptive analyses were done by determining means, medians, standard deviations and interquartile ranges (IQR). The frequency of a particular mutant or haplotype was calculated as the proportion of the specific mutant or haplotype among the total number of samples successfully analysed for this mutation. Categorical variables were compared using the Pearson chi-square or Fisher exact test, while continuous variables were compared by the Kruskall-Wallis test. The significance level (*P = 0.05*) was used in all analyses.

## Results

**Figure 1:**
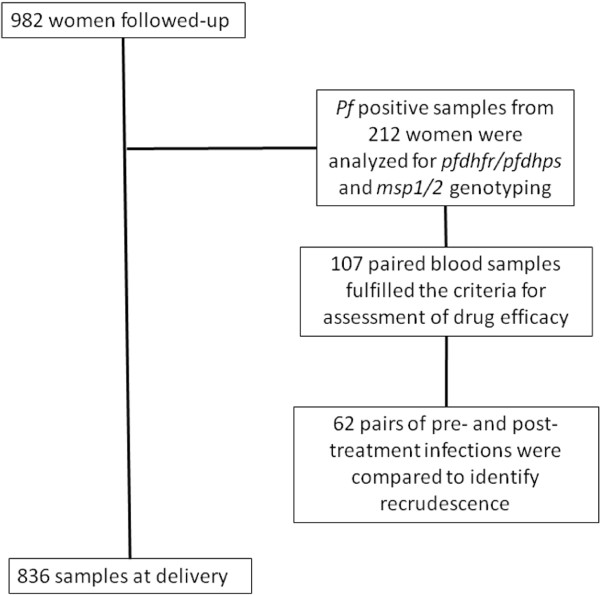
Flowchart diagram of the study.

In the STOPPAM study, a cohort of 982 women was enrolled and followed up, of whom 845 received two doses of SP, while 16, who were HIV-infected, received three doses. The first dose of SP-IPTp was given on average at 20 weeks of gestation, with the mean interval between the two doses of 35.4 days (SD = 9.9). A total of 836 women gave birth to live newborn and successfully completed the follow up. The parasite prevalence based on microscopical examination of blood smears was 17.0% at enrolment, falling to 4.1% one month after the first dose of SP-IPTp, and remaining stable until one month after the second SP-IPTp administration, before rising to 11.1% at delivery. Infections were mostly polyclonal with a multiplicity of infection (MOI) [IQR] of 3.6 [2.2-4.8]. The MOI at IPTp schedules was higher than that of recurrent infections following treatment (4.5 *vs* 2.7, *P = 0.04*). However no difference was seen in the MOI following the first or second dose (*P = 0.6*). Of the 191 women who were parasitized at baseline, 107 fulfilled the criteria to assess the *in vivo* drug efficacy (Figure 1).

### Trends of *pfdhfr* and *pfdhps* haplotypes throughout the antenatal clinic visits

**Figure 2:**
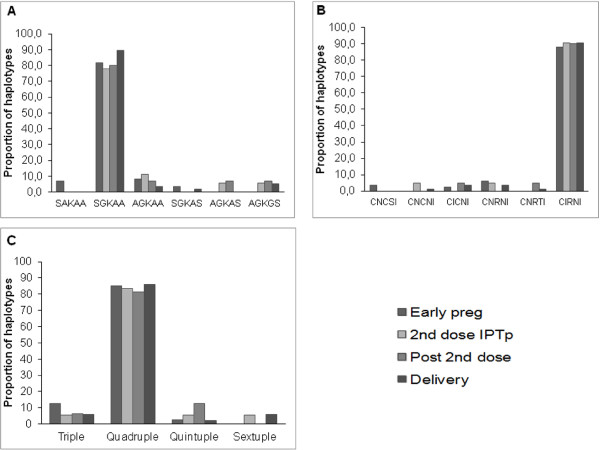
**Proportions of SNPs associated with SP resistance.** DNA from laboratory strains with known *pfdhfr* and *pfdhps* haplotypes (3D7, FRC3, K1, HB3, DD2, 7G8) were used as positive controls. Panel **A**: Proportion of SNPs associated with SP resistance on *pfdhps* gene: codons S436**A**, A437**G**, K540**E**, K613**S/T** and A581**G**. SGKAA, AGKAA are single mutant haplotypes while AGKGS represents double mutant. Mixed infections were excluded in the derivation of allelic haplotypes. Panel **B**: Proportions of SNPs associated with SP resistance on *pfdhfr gene*: codons N51I, C59R, S108N and I164L. Proportions are shown for isolates collected at different time-points. C**I**C**N**I, CN**RN**I and CN**RT**I are double mutant haplotypes while C**IRN**I represents triple mutant. Panel **C**: Proportions of combined *pfdhfr* and *pfdhps* allelic haplotypes. The combined analysis of both loci revealed that more than 80% of the parasite isolates had quadruple *pfdhfr*/*pfdhps* allelic haplotypes.

The prevalence of point mutations in the *pfdhfr* and *pfdhps* genes was determined in samples collected from 212 HIV-negative women; their general characteristics are shown in Table 1. At early pregnancy which corresponds to the day of the enrolment in the study, 88.0% of isolates consisted of triple mutant *pfdhfr* haplotypes C**IRN**I (C50, N51I, C59R, S108N and I164), 8.4% were double mutant haplotypes (C**I**C**N**I and CN**RN**I), while 3.6% were wild-type CNCSI (Figures 2A and 2B, Additional file 1: Table S1). This trend remained similar throughout the study period, as the majority of isolates (>80%) were of the triple mutant *pfdhfr* C**IRN**I haplotype, and there was no difference in prevalence according to time of sampling (*P = 0.7*). For the *pfdhps* gene*,* 81.7% of isolates at early pregnancy were single mutants S**G**KAA, 11.6% were double mutant A**G**KAA (8.3%) or S**G**KA**S** (3.3%), and 6.7% were of the wild-type haplotype SAKAA. As for *pfdhfr*, the haplotype distribution remained similar throughout the follow up, although haplotypes such as A**G**KA**S** and A**G**K**GS** were only found late in the follow up, at a prevalence of 6-7%. At codons *pfdhfr* I164 and *pfdhps* K540 only wild types were found. As expected, combining the two loci revealed that more than 80% of the parasite isolates were of the quadruple *pfdhfr*/*pfdhps* haplotypes (C**IRN**I-S**G**KAA and C**IRN**I-A**G**KAA) (Figure 2C). Although in low proportions (<10%), some less-mutated haplotypes, as well as more highly mutated quintuple or sextuple allelic haplotypes, were observed, with a tendency for more quintuple and sextuple haplotypes in late pregnancy (*P = 0.06*) (Figure 2C). The quintuple and sextuple mutants identified were mainly due to the combination of triple C**IRN**I *pfdhfr* mutant haplotype with the double (S**G**KA**S**, A**G**KA**S**) and triple (A**G**K**GS**) mutants of *pfdhps*, respectively.

**Table 1:** **Characteristics of the study population whose parasites were analysed by *pfdhfr, pfdhps, msp *1 (block 2), and *msp2 *genotyping**

	**Mean (SD)**	**Median**	**Range**
Mother’s age in years (n = 212)*	24.04 (5.98)	22	15-40
**Gestational age: weeks of pregnancy**			
At inclusion (n = 83)	18 (4.1)	17.4	4.0-25.0
At delivery (n = 88)	39.0 (1.9)	39.1	33.4-42.3
**First dose SP-IPTp**^§^**intake (n = 39)**	20.9 (3.2)	21	15-28.4
**Second dose SP-IPTp intake (n = 57)**	26.5 (3.4)	26.3	20-33.4
**Post second dose IPTp (n = 35)**	30.5 (3.5)	31	20-35.9
**Newborn’s birth weight in g (n = 212)**	2,881.2 (488.2)	2,900	1,100-4,050

Footnote: * Primigravidae (n = 63), Secundigravidae (n = 54), Multigravidae (n = 95).

^§^*SP-IPTp*, Sulphadoxine-pyrimethamine used for intermittent preventive treatment during pregnancy.

### Relationship between *pfdhfr* and *pfdhps* haplotypes and clinical outcome

There was no significant difference in parasite density among women infected with a particular parasite haplotype at any stage of pregnancy: 872 parasites/μl (105 quadruple infections) *vs* 4,625 parasites/μl (10 quintuple + sextuple infections, *P = 0.2*). In addition, the distribution of the various haplotypes detected during pregnancy and at delivery was not associated with either risk of placental infection (*P = 0.9*), maternal anaemia (*P = 0.5*), or low birth weight (*P = 0.*6). Maternal anaemia was defined as haemoglobin concentration under 11 g/dL and LBW as a birth weight <2,500 g.

### Identification and analysis of recurrent infections following IPTp uptake

**Figure 3:**
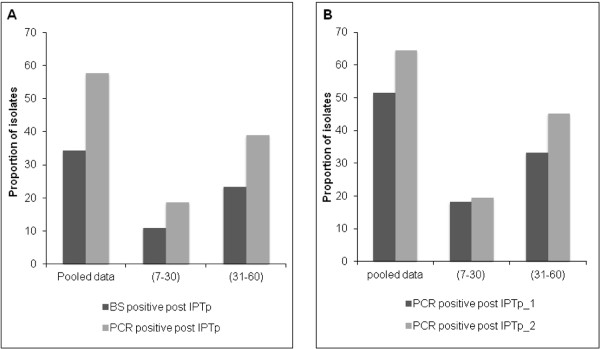
**Prevalence rates of recurrent infections. ***Plasmodium* parasites were counted against 200 leukocytes with a 100 × objective lens under oil immersion. Each qPCR reaction mixture contained 5 μL DNA template in a final volume of 20 μL, 10 μL of Master Mix (Applied Biosystem), both the genus-specific and *P. falciparum* specific primers and probes detection system (Plasmo/Pf) as described [24]. Panel **A**: Prevalence rates of recurrent infections assessed by microscopy (BS) and PCR. Data are shown as pooled (covering the two months following treatment) or split into first and second month (days 7–30 or 31–60). Panel **B**: Prevalence rates of recurrent infections assessed by PCR according to the IPTp dose received. Data are shown as pooled (covering the two months following treatment) or split into first and second month (days seven-30 or 31–60).

A total of 107 paired blood samples from women who had a positive blood smear at SP-IPTp administration (either first or second dose), and a blood sample available within a 60-day period following SP treatment were analysed by real-time PCR. Given the national guidelines of administration of two doses of SP-IPTp at an interval of at least one month, the follow-up time after the first dose of SP-IPT (29 days, range: 7–42) proved generally shorter than that after the second dose (37 days, range: 13–60, *P = 0.007*). Parasites were detected in 37 women (34.5%) by microscopy (blood smear = BS) and in 63 (59.0%) by PCR (Figure 3A, pooled data), of which approximately one third occurred in the first month after treatment (11% for BS and 19% for PCR), and two-thirds in the second month.

Segregating PCR-based data according to the sequence of the received dose of IPTp showed that the frequency of recurrence was higher after the second dose (64.6%) than after the first one (51.5%) (Figure 3B). Women were then split into those who cleared parasites during the follow up (BS-/PCR-), those in whom a submicroscopic parasitaemia persisted (BS-/PCR+) and those in whom a microscopic parasitaemia persisted (BS+/PCR+). No differential effect on birth weight was observed, but a trend for increased prevalence of maternal anaemia in women with persistent parasitaemia (*P = 0*.*07*) was noted. This trend was more explained by the extreme categories (BS-/PCR-) *versus* (BS+/PCR+) where maternal anaemia was observed in 52.5% *vs* 84.7%, respectively (*P = 0.03*) (Additional file 2: Table S2).

### Genotyping of recurrent infections following SP-IPTp uptake

**Figure 4:**
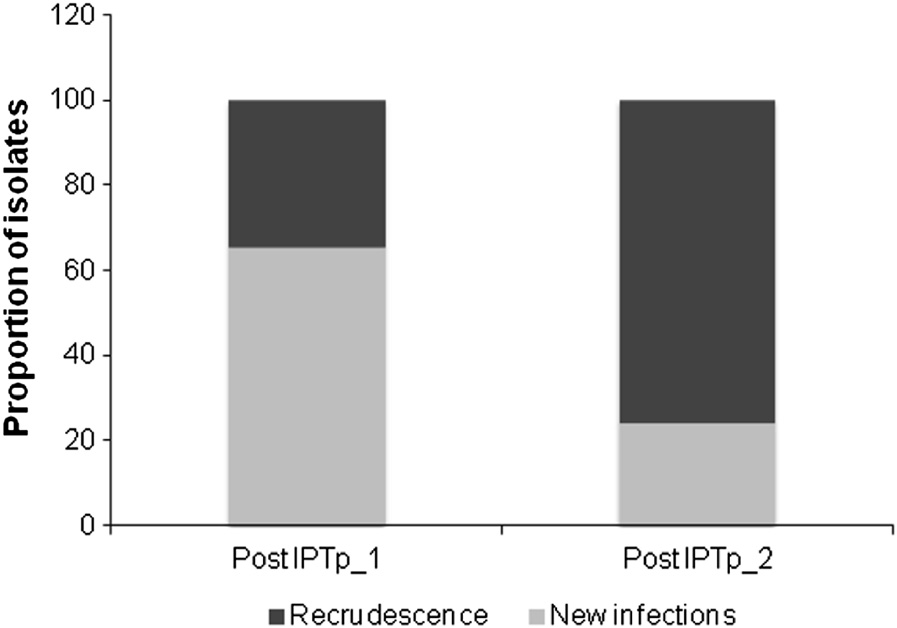
Proportions of recrudescent and new infections following SP treatment according to the sequence of IPTp uptake.

A total of 62 pairs of pre- and post-treatment infections identified by PCR were sequentially genotyped by amplifying the *msp2* and *msp1* (block 2) genes. Among these sample pairs, 52 (84.0%) were successfully genotyped. The mean parasitaemia of recurrent samples among unresolved infections, as estimated by real-time PCR, was lower than in those successfully genotyped (40 *vs* 1,000 parasites/μL, *P < 0.01*), explaining the failure to genotype some recurrences. Overall, recrudescent infections were more frequent than new infections (57.6% *vs* 42.3%). The frequency of recrudescence was markedly higher after the second SP-IPTp dose (75.9% of re-infections) than after the first (34.8%) (Figure 4).

**Figure 5:**
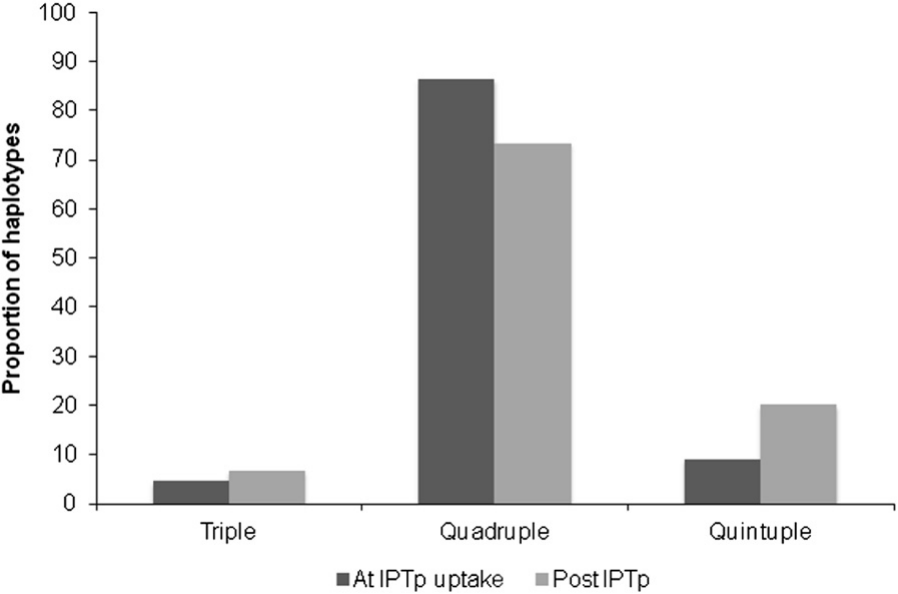
**Prevalence of *pfdhfr/pfdhps *haplotypes in consecutive parasite samples at baseline and after IPTp uptake.**

The *pfdhfr/pfdhps* haplotype analysis of this sub-group of consecutively collected parasite isolates showed a doubling in the frequency of quintuple mutants (from 9.1 to 20.0%) at the expense of quadruple mutants in post-treatment samples (*P = 0.06*), suggestive of a treatment-based selection effect (Figure 5).

## Discussion

Historically, the SP drug combination has been widely used in malaria-endemic countries of sub-Saharan Africa as a first-line drug to treat uncomplicated *P. falciparum* malaria. The emergence and widespread development of *P. falciparum* resistance to SP occurred over the last 30 years to reach the currently unacceptable level [31-37]. Characterization of the SNPs associated with SP resistance has provided an epidemiological tool to investigate the extent of resistance, as an alternative to the expensive and labour-intensive *in vivo* and *in vitro* drug sensitivity tests. Clearly, intensive past use of SP has been responsible for this spread of SP resistance, and to the upward trend in the prevalence rates of highly mutated *pfdhfr/pfdhps* parasites in West and East Africa [36,38]. The current study offered an excellent opportunity to investigate the level of penetration and the dynamics of the critical SNPs associated with SP resistance in a longitudinal analysis of *P. falciparum* isolates collected from pregnant women using SP-IPTp in Benin.

Based on blood smear, the highest prevalence rate of *P. falciparum* infections (17%) was observed at inclusion when women were screened for the first time, and had not yet received any anti-malarials, SP or other. Regardless of gestational age, the overwhelming majority of parasite isolates (~ 90%) carried the triple mutant *pfdhfr* (C**IRN**I) haplotype in association with the single *pfdhps* (S**G**KAA) mutation. A similarly high prevalence of *pfdhfr/pfdhps* mutations (85%) before any administration of SP-IPTp was reported in Southern Benin [22]. The current study provides an update of the situation six years after the withdrawal of SP as first-line anti-malarial drug, and three years after the study by Bertin [22], and suggests that the level of resistance has remained largely unchanged despite the reduced SP drug pressure on the parasite population. The identification of *pfdhps* codons 581 and 613 mutants is in agreement with published data, which indicates a comparatively low prevalence of these mutations [18]. Although, any significant relationship between the highly mutated haplotypes, such as the quintuple and sextuple mutants, with the occurrence of LBW and maternal anaemia was revealed, there is a tendency for the association of these mutants with higher peripheral parasite densities despite the limited sample size.

Of even greater concern, perhaps, is the fact that SP-IPTp failed to clear almost half of the infections overall, and that a large proportion of those occurred after completion of the second dose of SP-IPTp. In this study, the parasitological failure rate of SP, when analysed within a month, was 11 and 19% as determined by microscopy and PCR, respectively (Figure 2A). This is of course far lower than the failure rate of 73% reported among children less than five years old in the same region of Benin with a 28-day follow up [39], but entirely consistent with that reported in Tanzania where the failure rate at 28 days after SP treatment was 16% among pregnant women *versus* 80% in under five-year old children [40]. The substantially lower failure rates in pregnant women than in children is attributable to the stronger acquired anti-malarial immunity in adults, as well as possible pregnancy-specific differences in pharmacokinetics [41]. The extended follow up (60-day) in this study allowed detection of a greater number of recurrences, reaching 66% by PCR after the second dose of SP-IPTp. Clearly a longer follow up equates to less circulating drug and more exposure to re-infections. The longer follow up nevertheless also increased the ability to detect true recrudescence. Up to 76% of recurrent infections corresponded to true recrudescence. The fact that most of these infections were submicroscopic suggests that SP significantly reduced parasite densities, without complete clearance. The increasing parasite prevalence observed in the cohort during the third trimester, several weeks after the last SP-IPTp dose, is likely to be related to SP’s lack of efficacy. The effects of such submicroscopic infections at various gestational ages on the health of mother and foetus remain unknown. In this study, a clear relationship with birth weight, probably due to low sample size was unable to be demonstrated, but a trend towards more maternal anaemia was observed. A recent WHO review [21] concluded that, despite the high proportion of *P. falciparum* parasites carrying quintuple mutations, associated with *in vivo* SP resistance in some African countries, SP-IPTp remains effective in preventing the adverse consequences of malaria on maternal and foetal outcomes. A previously report showed that the incidence of malaria attacks, i e, symptomatic episodes, in the entire STOPPAM cohort reached a peak in the third trimester, long after the intake of the second dose of SP-IPTp [25]. This observation, allied to the current findings, indicates that the completion of the two-dose SP-IPTp regimen at a comparatively early gestational age left women susceptible to (re-)infection before delivery. Despite a decline from 17 to 4% in the prevalence of peripheral parasitaemia after treatment, the occurrence of recrudescent infections contributes to the rise in the prevalence later in pregnancy, as evidenced by the 11% prevalence found at delivery.

## Conclusions

These findings thus lend weight to the previous argument [42] for modification of the current SP-IPTp strategy to three doses instead of two in all mothers, as a third dose may contribute to maintaining low parasite densities and subdue adverse clinical outcomes. This is in line with a recently published meta-analysis from seven trials of 6,281 pregnancies in Africa, showing that the addition of a third SP dose was associated with an increase of birth weight and a lower risk of LBW than the standard two-dose regimen [43].

However, the high rate of recrudescence observed here highlights the lack of efficacy of SP in pregnant women. Under such conditions, changing the IPTp regimen using SP may seem to be based on pragmatic observations, but will only shift the problem as SP resistance will increase further. The urgency remains to find a more suitable alternative drug or a vaccine.

## Abbreviations

PAM: Pregnancy-associated malaria; PfIE: *Plasmodium falciparum*-infected erythrocytes; LBW: Low birth weight; SP: Sulphadoxine-pyrimethamine; IPTp: Intermittent preventive treatment; SNPs: Single nucleotide polymorphisms; DHFR: Dihydrofolate reductase; DHPS: Dihydropteroate syntase; OD: Optical density; MOI: Multiplicity of infection.

## Competing interests

The authors declare that they have no competing interests.

## Authors’ contributions

PD and NTN conceived and designed the study. AMO, YSSDT and JD performed the laboratory experiments. AMO, MA and NTN analysed the data. AMA contributed reagents/materials/analysis tools. AMO, JD, MA, AJFL, PD and NTN drafted and finalized the manuscript. The final manuscript was read and approved by all authors.

## Supplementary Material

Additional file 1: Table S1 Constructed haplotypes of *pfdhfr* and *pfdhps* detected during the follow-up. Click here for file

Additional file 2: Table S2 Clinical comparisons between women with and without persisting parasitaemia. Click here for file
