# Supplementary material for: High rates of parasite recrudescence following intermittent preventive treatment with sulphadoxine-pyrimethamine during pregnancy in Benin
Source: Malar J. 2013 Jun 10;12:195. doi: 10.1186/1475-2875-12-195 (PMC3686599; doi:10.1186/1475-2875-12-195)
Supplement: Additional file 1: Table S1 — Constructed haplotypes of pfdhfr and pfdhps detected during the follow-up. [file 1475-2875-12-195-S1.docx]

| *Pfdhps* haplotypes | | | | |  |
| --- | --- | --- | --- | --- | --- |
| Haplotype | Enrolment | 2nd dose | Post 2nd dose | Delivery | |
| SAKAA | 4 | 0 | 0 | 0 | |
| SGKAA | 49 | 14 | 12 | 52 | |
| AGKAA | 5 | 2 | 1 | 2 | |
| SGKAS | 2 | 0 | 0 | 1 | |
| AGKAS | 0 | 1 | 1 | 0 | |
| AGKGS | 0 | 1 | 1 | 3 | |
|  |  |  |  |  | |
| Total: | 60 | 18 | 15 | 58 | |
| Mixed | 9 | 2 | 2 | 4 | |
|  |  |  |  |  | |
|  |  |  |  |  | |
| ***Pfdhfr* haplotypes** | | | | |  |
| Haplotype | Enrolment | 2nd dose | Post 2nd dose | Delivery | |
| CNCSI | 3 | 0 | 0 | 0 | |
| CNCNI | 0 | 1 | 0 | 0 | |
| CICNI | 2 | 0 | 1 | 1 | |
| CNRNI | 5 | 1 | 0 | 3 | |
| CNRTI | 0 | 0 | 1 | 3 | |
| CIRSI | 0 | 0 | 0 | 1 | |
| CIRNI | 73 | 19 | 18 | 75 | |
|  |  |  |  |  | |
| Total: | 83 | 21 | 20 | 83 | |
| Mixed | 13 | 1 | 0 | 6 | |
|  |  |  |  |  | |
|  |  |  |  |  | |
| **Combined haplotypes** | | | | |  |
| dhps/dhfr | Enrolment | 2nd dose | Post 2nd dose | Delivery | |
| Triple | 5 | 1 | 1 | 3 | |
| Quadruple | 34 | 15 | 13 | 43 | |
| Quintuple | 1 | 1 | 2 | 1 | |
| Sextuple | 0 | 1 | 0 | 3 | |
|  |  |  |  |  | |
| Total | 40 | 18 | 16 | 50 | |

**Supplementary table 1 Constructed haplotypes of *pfdhfr* and *pfdhps* detected during the follow-up**
