# Supplementary material for: High rates of parasite recrudescence following intermittent preventive treatment with sulphadoxine-pyrimethamine during pregnancy in Benin
Source: Malar J. 2013 Jun 10;12:195. doi: 10.1186/1475-2875-12-195 (PMC3686599; doi:10.1186/1475-2875-12-195)
Supplement: Additional file 2: Table S2 — Clinical comparisons between women with and without persisting parasitaemia. [file 1475-2875-12-195-S2.docx]

**Supplementary table 2 Clinical comparisons between women with and without persisting parasitaemia.**

|  | Number | LBW | ANEMIA | Mean birth weight (CI 95%) | |
| --- | --- | --- | --- | --- | --- |
| PCR- BS- | 16 | 3 | 10 | 2863.44 | [2560.52 ; 3166.35] |
| PCR+ BS- | 25 | 4 | 18 | 2955 | [2771.14 ; 3138.86] |
| PCR+ BS+ | 19 | 4 | 18 | 2822.37 | [2535.59 ; 3109.15] |

**Footnote:** The overall comparison highlights a tendency for anaemia (p = 0.07), but the difference is mainly due to two extreme groups PCR- BS-vs PCR+BS (p = .03). No such difference was observed with birth weight.
